# Auricular acupressure for myopia prevention and control in children and its effect on choroid and retina: a randomized controlled trial protocol

**DOI:** 10.1186/s13063-021-05334-1

**Published:** 2021-06-07

**Authors:** Rong Han, Xie-he Kong, Feng Zhao, Yan-ting Yang, Xiao-qing Dong, Li Zeng, Zhi Chen, Yue Zhao, Guang Yang, Jue Hong, Xing-tao Zhou, Xiao-peng Ma

**Affiliations:** 1 Shanghai University of Traditional Chinese Medicine, Shanghai, 201203 China; 2 Shanghai Research Institute of Acupuncture and Meridian, Shanghai, 200030 China; 3 Department of Ophthalmology, Shuguang Hospital, Shanghai University of Traditional Chinese Medicine, Shanghai, 201203 China; 4 Department of Ophthalmology, Eye and ENT Hospital of Fudan University, Shanghai, 200031 China

**Keywords:** Myopia prevention and control, Auricular acupressure, Randomized clinical trial, Choroid, Retina

## Abstract

**Background:**

Nowadays, because of the increasing incidence, the prevention and control of myopia has become an urgent issue. In China, auricular acupressure has been commonly used in the clinical treatment of myopia in children, but the exact effectiveness remains unproven. The purpose of this trial is to observe the efficacy of auricular acupressure in myopia prevention and control, as well as its effect on the choroidal and retinal thickness.

**Method/design:**

A total of 480 subjects at 8–9 years old will be randomized in a 1:1 ratio to an intervention group versus a control group. The intervention group will receive auricular acupressure for 12 months, while the control group will be taken as a blank control. The primary and secondary outcomes will be measured at baseline, and again at 3, 6, 9, and 12 months after recruitment. The myopia incidence (spherical equivalent ≤ − 0.50 D) and the mean change of spherical equivalent will be taken as the primary variables; the secondary outcome measures include axial length, uncorrected visual acuity, and choroidal and retinal thickness.

**Discussion:**

This trial aims to evaluate the effectiveness of auricular acupressure for myopia prevention and control with objective evidence and to preliminarily explore the plausible mechanism and provide reference for adopting this approach to retard the onset and control the progression of myopia.

**Trial registration:**

Chinese Clinical Trial Registry ChiCTR2000038456. Registered on September 23, 2020.

**Supplementary Information:**

The online version contains supplementary material available at 10.1186/s13063-021-05334-1.

## Research background

Myopia is a condition in which the resting eye focuses the parallel rays in front of the retina due to excessive axial elongation or refraction. Large-scale epidemiological survey conducted in China indicated that the prevalence of myopia is now 70–85% in students at the age of 17 to 18 [1–3], being extremely higher than that of 25.7% seen before 2001 [4]. In East Asia, myopia is found to progress rapidly, especially in school children. Although East Asian countries have the highest prevalence, an increasing myopic shift is also observed in Australia [5], Northern Ireland [6, 7], New Delhi [8, 9], and other regions of the world. By 2050, it is estimated that nearly 5 billion people will be affected by myopia at the global scale [10]. Hence, the rapidly increasing prevalence of myopia is of worldwide concern in the twenty-first century. Early-onset myopia may progress to high myopia, which predisposes the patients to a series of sight-threatening pathological consequences, such as retinal detachment and macular degeneration [11]. Therefore, a strategy to postpone the age of myopia onset has become an important issue to be solved urgently.

Currently, the interventions for myopia are under four categories [12]: optical, pharmacological, surgical, and environmental (behavioral). Increased time spent outdoors is the only intervention known to reduce the onset of myopia, but it requires at least 14 h to neutralize the risk of parental myopia [13], which is nearly a mission impossible considering the intense education in Chinese children. Because both, myopia onset and myopia progression, depend on or are associated with axial elongation, some interventions known to slow progression can also be applied to pre-myopes. Orthokeratology treatment is limited to children over 8 years old in China and its efficacy seems negatively correlated with the age of the Chinese children [14], so younger children may not benefit from it and its related infection risk also cannot be ignored. In recent years, the effect of 0.01% atropine eye drops in slowing myopic progression has been shown in different studies from various countries [15–17], but its effect on axial length elongation remains controversial [15, 16]. A higher concentration atropine has been intended for children who showed poor response to 0.01% atropine. However, greater accommodative amplitude loss and pupil dilation [18] will be induced, not favorable in terms of long-term safety. Surgical interventions such as posterior scleral reinforcement aim at stabilizing highly myopic eyes, which are not common strategies for myopia prevention. Yet, no ideal therapy is available for preventing the onset of myopia in China.

Acupuncture-moxibustion is one major part of traditional Chinese medicine (TCM). Acupuncture treatment can produce certain effect in reducing myopia in kids [19]. Nevertheless, acupuncture requires high operation skills and most children are afraid of needling, which make it difficult for large-scale promotion. As another acupuncture-moxibustion treatment branch, auricular acupressure has been commonly used to treat ocular diseases, especially myopia in children, because it is simple-to-operate, non-invasive, and less likely to cause side effects [20]. Previous studies [21–23] have shown that auricular acupressure can effectively slow down the progression of myopia, but these studies are rather limited by poor research methods, such as small sample size and short intervention time, which have weakened this conclusion. To today, there is no high-quality research studying how well auricular acupressure works to prevent myopia or slow down its progression, and thus it is of great clinical value to conduct such a study.

Axial elongation is one of the main anatomical changes with the increase of diopter. The stretching of axial elongation will make the choroid and retina thinner [24, 25]. Retina serves as the center in refractive regulation, in which photoreceptors, nerve fibers, and other structures are closely related to the onset and development of myopia [26]. Choroid provides oxygen and nutrition to retina and adapts the retina to the focal plane of the eye by changing its thickness [27, 28]. Moreover, thicker choroid will hinder the transfer of metabolic growth factors to sclera, slowing down the synthesis of scleral matrix and the axial growth [29]. In addition, choroid responds faster than the eye’s axis. Therefore, observing choroidal thickness is helpful to observe the development of early-stage myopia and explore its mechanism [30, 31].

This trial is supposed to evaluate the effectiveness of auricular acupressure in the prevention and control of myopia in children by observing the myopia incidence (MI), changes in spherical equivalent (SE), axial length, and uncorrected visual acuity and to explore the pertinent action mechanism by detecting the changes in choroidal and retinal thickness. The results of this study will provide clinical evidence for using auricular acupoints to prevent and control myopia in children.

## Method/design

### Design of the trial

This parallel, randomized, blank controlled prospective clinical trial will be carried out in the Eye and ENT Hospital of Fudan University and Shuguang Hospital Affiliated to Shanghai University of Traditional Chinese Medicine, two top-rank hospitals in China. The subjects will be randomized in a 1:1 ratio to an intervention group versus a control group. The intervention group will receive auricular acupressure for 12 months, while the control group will not receive any interventions. A series of optical examinations will be performed at baseline, and 3, 6, 9, and 12 months after recruitment. The whole flow of the trial is illustrated in Figure S1 and the schedule is shown in Table 1. During the study, the subjects will be asked to keep a journal to record their compliance. The research staff will inform each subject of the details of the study before intervention, and all the subjects and their guardians will provide written consent before the treatment starts and they will all be given sufficient time to reach the final decision.

**Table 1:** Schedule of treatment assessments and data collection

	Enrollment and baseline	Study period					
		Allocation	Post-allocation				Close-out
Time point (month)	-	0	3	6	9	12	
Informed consent	×						
Inclusion/exclusion	×						
General information	×						
Random allocation		×					
Intervention (auricular acupressure)		×	×	×	×	×	
SE (after cycloplegia)	×		×	×	×	×	
Axial length	×		×	×	×	×	
Uncorrected visual acuity	×		×	×	×	×	
Corneal curvature	×						
Retina thickness	×		×	×	×	×	
Choroid thickness	×		×	×	×	×	
Outdoor light exposure			×	×	×	×	
Near work			×	×	×	×	
Concomitant medication	×		×	×	×	×	
Adverse events and other unintended effects			×	×	×	×	
Compliance			×	×	×	×	
Completion summary							×

This trial will follow the Declaration of Helsinki (version 2013) and has been approved by the Ethics Committee of Shuguang Hospital Affiliated to Shanghai University of Traditional Chinese Medicine (approval No. 2020-801-08-01) and registered at www.chictr.org.cn (ChiCTR2000038456).

### Subjects

#### Inclusion criteria

Those who are willing to participate in the trial and meet the following conditions will be included: ages 8 to 9 years old; SE (after cycloplegia), − 0.5 D to + 0.5 D; uncorrected visual acuity ≥ 0.8; corneal curvature, 40 D–46 D; with good compliance; and the subject and his/her guardians signed the informed consent form.

#### Exclusion criteria

Those who meet any of the following conditions will be excluded: combined with other eye diseases (cataract, congenital retinal disease, strabismus, amblyopia, etc.) or systemic diseases; with active eye diseases or had a history of eye surgery; those who have used atropine, orthokeratology lens, or other myopia prevention and control methods; skin lesion at the to-be-treated auricular acupoints or sensitive to the ear adhesive; subjects who cannot cooperate to complete the treatment; and the guardian has unreasonable expectation.

#### Elimination criteria

Those who do not meet the inclusion criteria or match the exclusion criteria but were mistakenly included and have no post-randomization observation.

#### Suspension criteria

Serious adverse events occur and the observation cannot continue; severe complications or special physiological changes occur, and the study cannot continue.

#### Dropout criteria

Despite the reason, the subject or guardian is unwilling or impossible to continue and proposes to withdraw from the study; those who did not propose to withdraw from the study but fail to complete the whole treatment.

#### Recruitment

We will recruit most of the subjects by placing posters in schools, hospitals, and communities, while the rest will be recruited at the outpatient. All the recruitment advertisements will only be published after the trial was approved by the ethics review committee. The respondents will contact the research staff by telephone, WeChat, or in outpatient visit. The researchers will screen the subjects according to the inclusion and exclusion criteria, and collect a part of the baseline data during the screening. The recruitment process will continue until the number of recruits reaches the expected standard. The recruitment process is shown in Figure S1.

#### Sample size

So far, there is no high-quality randomized controlled trial (RCT) studying the sole use of auricular acupoints in reducing the MI. Increasing outdoor time is the main method well recognized in the prevention of myopia. Therefore, the preventive effect of outdoor activities is used to estimate the efficacy of auricular acupoints. The MI in the intervention group 8.41%/year, and that in the control group is 17.65%/year [32]. When we set α = 0.05, test efficiency 1 – β = 0.80, and the ratio between the two groups 1:1, the calculated required sample size is 204 for each group according to the following equation, that is 408 in total. Then, when the dropout is set at 15% or less, a total of 480 participants should be recruited in this study.

$$ n=\frac{{\left({\mathrm{Z}}_{1-\alpha }+{\mathrm{Z}}_{1-\beta}\right)}^2}{{\left({\pi}_r-{\pi}_c\right)}^2}\left[{\pi}_r\left(1-{\pi}_r\right)+{\pi}_c\left(1-{\pi}_c\right)\right] $$

$$ {\pi}_r= MI\ \mathrm{in}\ \mathrm{the}\ \mathrm{intervention}\ \mathrm{group} $$

$$ {\pi}_c= MI\ \mathrm{in}\ \mathrm{the}\ \mathrm{control}\ \mathrm{group} $$

### Randomization and grouping

SPSS version 25.0 will be used to generate random numbers and random distribution table. According to the random sequence, the paper marked with intervention group or control group will be put into a sealed opaque envelope. The subjects will be randomized into an intervention group or a control group at 1:1. The randomization process will be completed by a third party unrelated to the study. Only after the informed consent of the subjects has been obtained and the subjects have completed the baseline data collection, the allocation personnel can inform the acupuncturists of the allocation assignment.

### Blinding

Due to the unique feature of the intervention, we can only blind the examiners, data processors, and statisticians but not the interveners or subjects. Before each examination, subjects in the intervention group will be asked to take off the auricular adhesives, which will be checked by the personnel in charge of randomization. During the whole study, the subjects and other researchers are not allowed to disclose the grouping to examiners, data processors, and statisticians. Only when serious adverse events occur and the researchers believe that it is necessary to know the intervention to take effective measures, can the blinding rule be broken. The researchers will be trained strictly before the trial begins.

### Intervention

#### Intervention group

Subjects in the intervention group will be treated with auricular acupressure for 12 months. The product used for auricular acupressure is Wangbuliuxingzi (vaccaria seed) auricular adhesives (Shanghai Taicheng Science and Technology Development Co., Ltd., registration No. Huxuxiebei 20160002), and the product information is shown in Figure S2. According to the clinical experience of our research group and the effective prescription published before [20, 33], six acupoints [eye (LO_5_), liver (CO_12_), kidney (CO_10_), heart (CO_15_), spleen (CO_13_), and shenmen (TF_4_)] will be selected as the auricular acupoint protocol in this study. The localization of the acupoints refers the Nomenclature and Location of Auricular Acupoints [34] stipulated by the World Federation of Acupuncture-Moxibustion Societies (WFAS) in 2013. The anatomical locations are listed in Table 2 and illustrated in Figure S3.

**Table 2:** Location of the auricular acupoints

Acupoint	Location
Eye (LO_5_)	In the center of the anterolateral surface of the ear lobe
Liver (CO_12_)	In the posteroinferior part of the cymba conchae
Kidney (CO_10_)	In the cymba conchae below the posterior region of the inferior antihelix crus
Heart (CO_15_)	In the center of the depression of the cavum conchae
Spleen (CO_13_)	In the posterior upper part of concha cavity
Shenmen (TF_4_)	In the upper part of the posterior 1/3 of the triangular fossa

Operation is as follows: After standard sterilization for the auricular acupoints with 75% alcohol, the auricular adhesives are attached to the acupoints with the pellets precisely targeting the acupoints, eye (LO_5_), liver (CO_12_), kidney (CO_10_), heart (CO_15_), spleen (CO_13_), and shenmen (TF_4_), and pressed tightly. This process will be operated by professional acupuncturists after unified training. The guardian is asked to press the adhesives once every day in the morning (6:30 am–7:30 am), after school (4:00 pm–5:00 pm), and before bed time (9:00 pm–10:00 pm). A good adherence is defined as “completing at least 5 times of acupressure operation during two consecutive days (required to do 3 times a day)”. Each acupoint should be pressed 20 times each session, and the press should be causing distending and aching sensation but within the kid’s tolerance. Every night, the guardian should record the completion status in the diary card, and send the diary card photos to the researcher through WeChat every week. One ear is treated each time (to be alternately at each visit), and the auricular adhesives are changed by the acupuncturist each week. Since the adhesives may fall off due to exercise, bathing and other unexpected conditions, the researcher would send the auricular acupoint atlas and the video on auricular point sticking to the guardian after recruitment, so as to guide the guardian to re-apply the plaster to the original spot after the adhesive falls off. If skin abrasion or swelling occurs at the treated spots during, the guardian needs to contact the acupuncturist immediately to deal with it, which will be recorded in the case report form (CRF).

#### Control group

The subjects in the control group will not receive any interventions for myopia prevention or control within 12 months, but only go to the hospital to check the relevant optical indicators at the required time points.

### Outcome observation

Subjects in both intervention group and control group will be measured for SE, axial length, uncorrected visual acuity, retinal, and choroidal thickness at baseline, and 3, 6, 9, and 12 months after recruitment. All the researchers involved in data evaluation were trained strictly before the start of the trial.

#### Primary outcome measures

The primary outcome measures include MI and SE.

The measurement of SE will be carried out with Nidek ARK-1 refractometer produced by Nidek, Japan. Before measurement, the subjects were given one drop of 0.5% proparacaine hydrochloride eye drops (Alcaine), one drop of 1% cyclopentolate hydrochloride eye drops (CYCLOGYL) 1 min later, and one drop of 1% cyclopentolate hydrochloride eye drops (CYCLOGYL) 5 min later, to induce cycloplegia, getting ready for automatic optometry 30 min later. The measurement will be performed three times successively. Make sure that the maximum-minimum difference in the spherical and cylindrical diopters should be no more than 0.25 D, respectively, and the average value of the three measurement results will be recorded. The SE is defined as spherical diopter plus 1/2 cylindrical diopter. According to existing reports and clinical routines [35], we define myopia as SE ≤ − 0.50 D.

#### Secondary outcome measures

The secondary outcome measures include the eye’s axial length, uncorrected visual acuity, and choroidal and retinal thickness.

Measure the axial length using IOL-Master (Carl Zeiss, Germany): Make sure the examinee takes a comfortable position. Adjust the height of the instrument according to the height of the examinee, and instruct him/her to fix the mandible and forehead on the jaw and frontal brackets, and adjust the eyes’ position. Ask the examinee to keep their eyes still by looking at the fixation lamp. The axial length will be measured three times and the mean value will be obtained.

The uncorrected visual acuity will be tested using the international standard visual acuity examination charm at a 5-m distance under the same lighting condition. From the optotype 4.0, move down line by line if the participant can see it clearly; if not, then check upward line by line. The participant will be given maximum 5 s to identify each optotype. From the line 4.0 to 4.5, move up one line when one optotype in the same line is misread; from line 4.6 to 5.0, two misreads allowable; from line 5.1 to 5.3, the number is three. The best visual acuity will be recorded if the participant successfully tells all the optotypes; if not, the final visual acuity will be considered the last line successfully identified. The results will be converted into Log-MAR for statistical analysis.

Measure the choroidal and retinal thickness using optical coherence tomography (OCT, RS-3000; NIDEK, Japan): Prior to OCT examination, the examiner should describe the examination process and precautions to the subject, and start the examination after obtaining the consent. The examinee stays in a sitting position. Adjust the height of the instrument according to the height of the examinee, and instruct the subject to fix the mandible and forehead on the brackets, and adjust the eyes’ position. The retina will be scanned in a cross-sectional manner in the range of 6 mm × 6 mm with macular fixation module, and the scanning center coincides with the fovea. The OCT built-in software program automatically detects and displays layers of retinal and choroid. Each layer is divided into three concentric circles: 1 mm central fovea, 1–3 mm parafovea, and 3–6 mm perifovea. The parafovea and perifovea can be further divided into 4 regions: upper, lower, nasal, and temporal. So there are a total of nine regions to measure the thickness of retina and choroid.

### Data collection and management

The collection of all baseline data and general information of each subject will be completed within 1 week before the start of the intervention. The general data include gender, age, school grade, number of myopic parents, past history, and medication history. The baseline data include spherical equivalent, axial length, uncorrected visual acuity, corneal curvature, and choroidal and retinal thickness. At 3, 6, 9, and 12 months after recruitment, the SE, axial length, uncorrected visual acuity, and choroidal and retinal thickness will be measured again to observe the changes. The data collection timeline is shown in Figure S4.

The information of each subject will be recorded in the CRF and the electronic database ResMan, with codes and abbreviations used instead of their names to protect privacy. The data in CRF form and ResMan will be independently input and checked by two third-party data administrators unrelated to the test to ensure the accuracy. If the subject requests to withdraw from the trial, the eye condition of the subject will be evaluated as quickly as possible. For suspicious data, the data manager will conduct data recheck query (DRQ) and give feedback to the researcher. The researcher should reply as soon as possible for the doubtful data, and the data manager will modify, confirm, and input the data according to the researcher’s reply. All paper and electronic documents will be kept, and the data printed on thermal paper will be photographed. Therefore, if readers or reviewers have any questions, they can certainly contact the authors for the original data.

### Statistical analysis

SPSS version 25.0 will be used for data processing in this trial. Only ocular parameters of the right eye will be included for statistical analysis. Analyses will be performed based on a modified intention-to-treat principle and involve only subjects who have both a baseline outcome measurement and at least one post-randomization observation. Last-observation-carried-forward approach will be used to replace missing data.

The measurement data will be expressed as means ± standard deviation or median (interquartile interval), and the counting data will be described by the number of cases (percentage). Fisher’s exact test or Pearson’s chi-square test will be used for trial group comparisons of 1-year cumulative MI. Generalized estimating equations (GEE), with one within-subject factor (time), one between-subject factor (intervention), and their interactions, will be used to compare changes in spherical equivalent, axial length, uncorrected visual acuity, and choroidal and retinal thickness. The covariates in the GEE models include the corresponding baseline measures, age, gender, number of myopic parents, outdoor light exposure time, and near-work time. All the tests of effects will be conducted at a two-sided alpha level of 0.05.

### Safety evaluation

In case of any adverse reaction during the treatment, the subject should contact the researchers immediately. The researchers will diagnose and deal with the adverse reactions, making decision whether it is necessary to stop the test or provide corresponding compensation, and judging if the adverse reaction is caused by auricular acupressure. The adverse reactions included topical ear pain, swollen ear skin, ear skin lesion, palpitation, or nausea. All adverse events should be recorded in the CRF for later analysis.

### Trial monitoring

The Data and Safety Monitoring Committee (DSMC) would be established before the trial starts. It is composed of experts on trial design and statistics to supervise the implementation, data collection, safety and privacy protection of subjects, and make suggestions on the modification of the trial. If there are any issues that may affect the progress of the trial, the interests or safety of patients, the protocol needs to be formally modified. Such amendments will be agreed upon by DSMC, approved by the Ethics Committee prior to implementation. When 50% of the patients were randomized and completed a 6-month follow-up, an interim analysis of the main indicators was performed. The interim analysis was performed by an independent third-party statistician blind to treatment allocation. The statistician will report to DSMC. DSMC will provide accessible access to all data, discuss the results of the interim analysis, and decide whether to continue the trial.

## Discussion

The continuing increase of myopia incidence and its tendency affecting younger populations have made myopia a critical public issue in China. TCM has a long history in the treatment of eye diseases. The prevention and treatment methods include Chinese medication, acupuncture, moxibustion, and massage. Currently, the common method for prevention and control of myopia in kids is the eye exercises, a set of massage method based on the meridian theory of TCM and sports medicine. By massaging the acupoints around the eyes, it can regulate qi and blood flow in the eyes, so as to relax the muscles and improve blood circulation of the eyes [36]. Recent study showed that compared with no eye exercises, 2 years of high-quality eye exercises slowed down the progression of myopia by 0.15 D, which, however, had no significant difference [37]. Auricular acupoint therapy refer to the method of using corresponding tools to stimulate auricular acupoints to diagnose and treat diseases under the guidance of TCM and acupuncture-moxibustion theories and modern medical theory. Research showed that auricular acupoint combined with other methods effectively reduced the development of myopia [22, 23], but its prevention and treatment effect on myopia needs more evidence to clarify. This trial will objectively evaluate the prevention and control effect of auricular acupoints on myopia by measuring SE, axial length, and uncorrected visual acuity. It will also explore the possible mechanism by observing the changes in choroidal and retinal thickness, providing reference for clinical application of auricular acupoints in prevention and control of myopia in children, and contributing clinical evidence for seeking simple and effective methods for myopia prevention and treatment in the future.

Differential diagnosis and treatment is one unique characteristic in TCM. In the actual diagnosis and treatment process, doctors will carry out individually tailored treatment based on each patient’s own situation. Therefore, how to select the acupoints in clinical acupuncture-moxibustion trials is a problem faced by acupuncture researchers worldwide. In this protocol, same auricular acupoint prescription will be used in all subjects in the intervention group. This fixed prescription is based on the comprehensive consideration of the feasibility and quality control of the study, and it is suitable for the prevention and control of various types of myopia. In TCM theory, myopia is mainly due to imbalanced function of the heart, liver, spleen, and kidney, leading to a lack of source transforming into essence and qi, in which the eyes cannot obtain enough nutrition, so that the physiological function of the eyes is damaged [38, 39]. In the auricular acupoint prescription, eye (LO_5_) is selected according to the disease location, and stimulating this point can unblock qi and blood flow in the eyes; since the liver and kidney share the same origin according to TCM theory, liver (CO_12_) and kidney (CO_10_) can be used together to regulate and reinforce the two organs, nourish yin and supplement essence, maintain blood and brighten the eyes; heart (CO_15_) can adjust heart qi and soothe the mind, so that qi and blood will be sufficient to go up and nourish the eyes; spleen (CO_13_) can strengthen the spleen function and supplement qi, and help with the production and transformation of qi and blood, so that the essence and qi can travel upward to nourish the eyes; shenmen (TF_4_) functions to sedate and calm the mind, so it can be used together with liver (CO_12_), kidney (CO_10_), heart (CO_15_), and spleen (CO_13_) to strengthen the effect on mind. The combination of these acupoints work to regulate and tonify the internal organs, conserve blood and replenish qi, activate blood circulation, and dredge collaterals, so that qi and blood can fully nourish the eyes to improve vision.

The drawback of this trial is that we did not use sham intervention to blind the subjects. Since soreness caused by auricular acupressure is well recognized by people in China and stimulation of the auricle other than the areas of our acupoints such as helix can also cause a physiological response [40], neither using the seed-free acupoint plaster or sham-acupoint plaster with seed is an ideal sham intervention. However, evaluating the clinical efficacy by quantitative, objective indices mitigates the risk of bias.

## Supplementary Information

**Additional file 1: Figure S1**

**Additional file 2: Figure S2**

**Additional file 3: Figure S3**

**Additional file 4: Figure S4**

**Additional file 5.** All items from the World Health Organization Trial Registration Data Set

## Data Availability

The statistics and materials used in this trial can be obtained by contacting researchers, and the original data will be disclosed 6 months after the completion of the trial.

## Abbreviations

CRF: Case report form
DRQ: Data recheck query
DSMC: Data and Safety Monitoring Committee
MI: Myopia incidence
OCT: Optical coherence tomography
RCT: Randomized controlled trial
SE: Spherical equivalent
TCM: Traditional Chinese medicine
WFAS: World Federation of Acupuncture-Moxibustion Societies
