# Supplementary figures and images for: Auricular acupressure for myopia prevention and control in children and its effect on choroid and retina: a randomized controlled trial protocol
Source: Trials. 2021 Jun 7;22:387. doi: 10.1186/s13063-021-05334-1 (PMC8186104; doi:10.1186/s13063-021-05334-1)

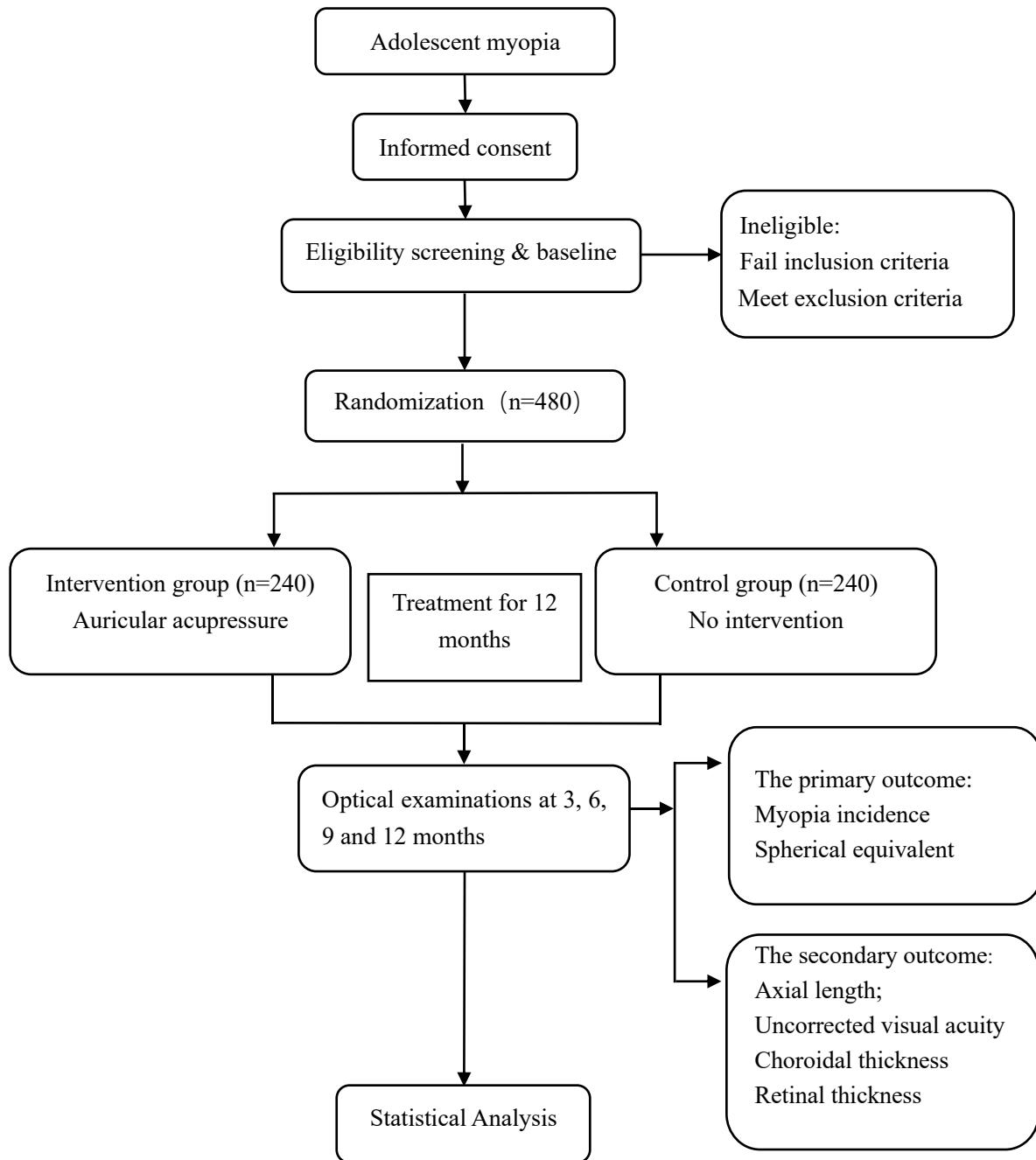

Supplement: Supplementary file 1 — Additional file 1: Figure S1 [file 13063_2021_5334_MOESM1_ESM.pdf]

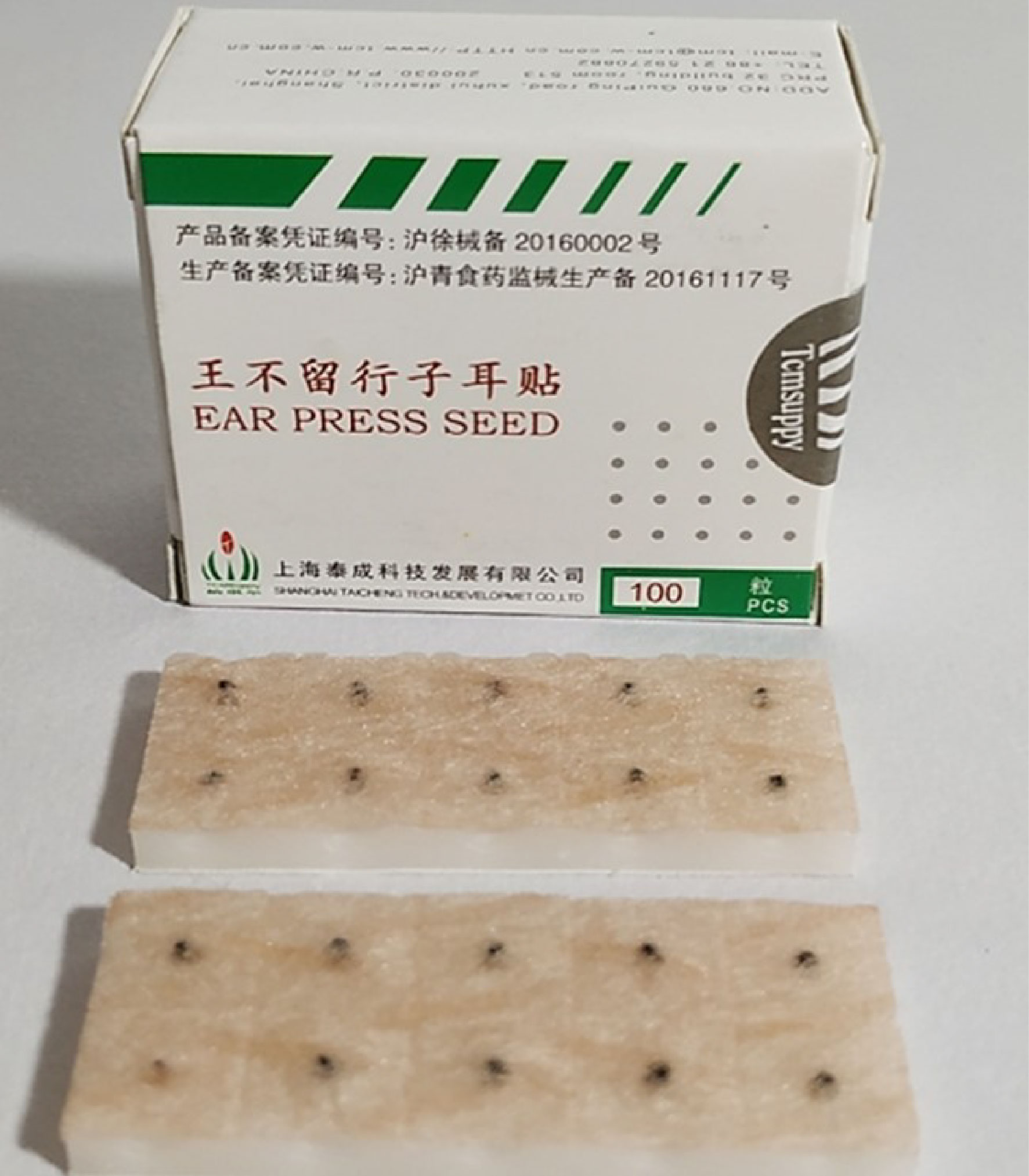

Supplement: Supplementary file 2 — Additional file 2: Figure S2 [file 13063_2021_5334_MOESM2_ESM.jpg]

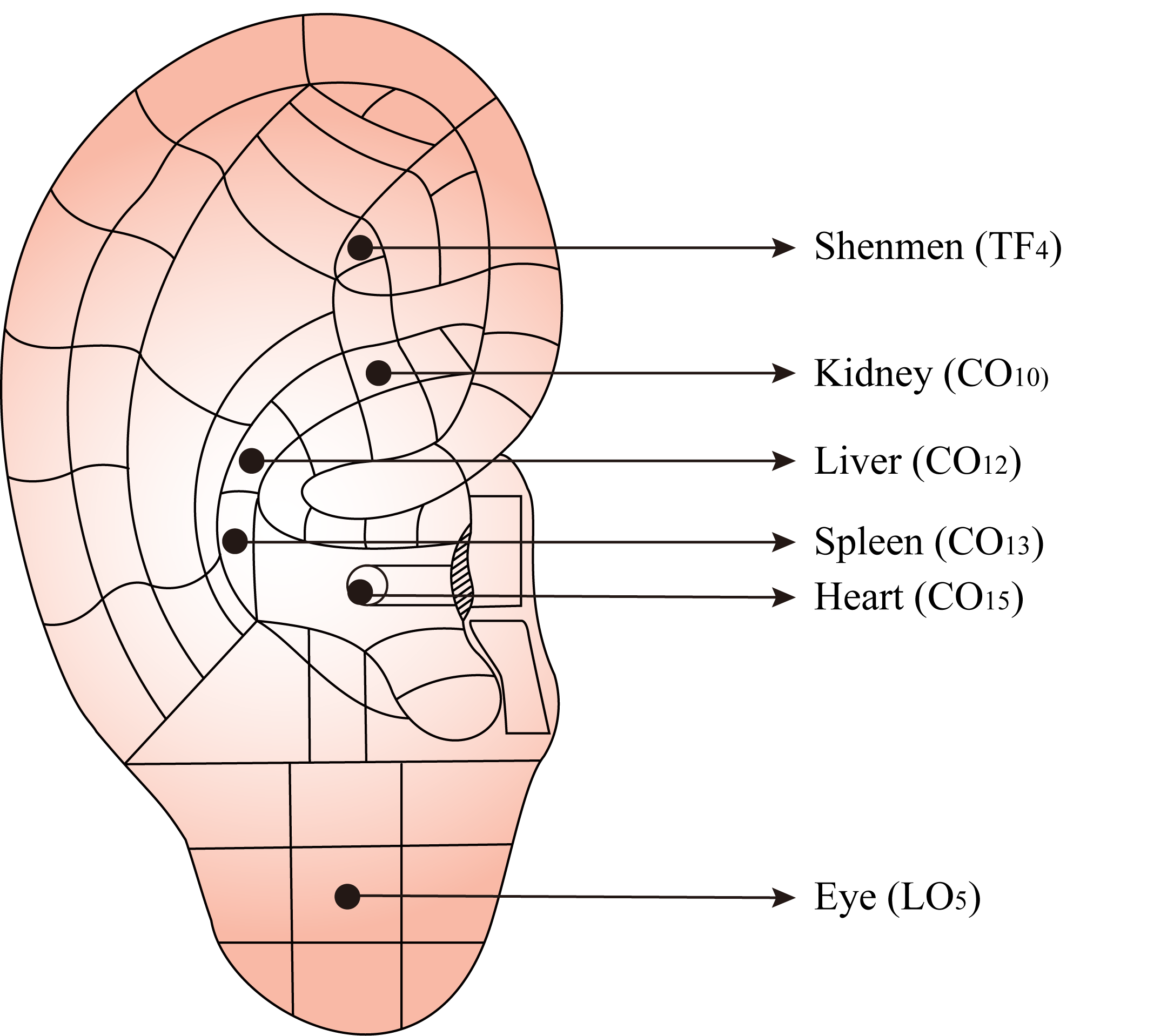

Supplement: Supplementary file 3 — Additional file 3: Figure S3 [file 13063_2021_5334_MOESM3_ESM.tif]
