# Supplementary material for: Auricular acupressure for myopia prevention and control in children and its effect on choroid and retina: a randomized controlled trial protocol
Source: Trials. 2021 Jun 7;22:387. doi: 10.1186/s13063-021-05334-1 (PMC8186104; doi:10.1186/s13063-021-05334-1)
Supplement: Supplementary file 4 — Additional file 4: Figure S4 [file 13063_2021_5334_MOESM4_ESM.doc]

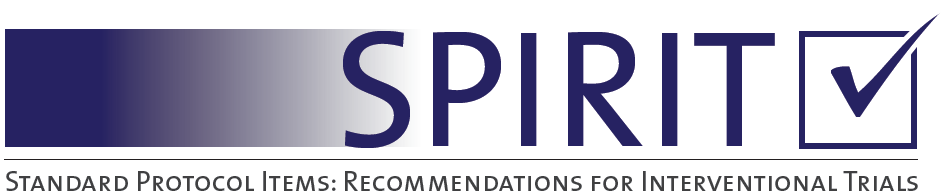


SPIRIT 2013 Checklist: Recommended items to address in a clinical trial protocol and related documents*

| Section/item | ItemNo | Page number (Line number) |
| --- | --- | --- |
| **Administrative information** | | |
| Title | 1 | Page1 (Line1-3) |
| Trial registration | 2a | Page2 (Line33-35) |
|  | 2b | Additional Files 5 |
| Protocol version | 3 | Page18 (Line352-353) |
| Funding | 4 | Page19 (Line363) |
| Roles and responsibilities | 5a | Page1 (Line4-12) |
|  | 5b | Page19 (Line364) |
|  | 5c | Page19 (Line365-366) |
|  | 5d | Page19 (Line370) |
| Introduction |  |  |
| Background and rationale | 6a | Page2-4 (Line38-92) |
|  | 6b | Page3 (Line51-77) |
| Objectives | 7 | Page4 (Line87-92) |
| Trial design | 8 | Page5-6 (Line94-109) |
| Methods: Participants, interventions, and outcomes | | |
| Study setting | 9 | Page5 (Line95-97) |
| Eligibility criteria | 10 | Page6-7 (Line112-123) |
| Interventions | 11a | Page9-11 (Line172-204) |
|  | 11b | Page7 (Line124, Line127, Line130) |
|  | 11c | Page10 (Line188-193) |
|  | 11d | Page7 (Line120-123) |
| Outcomes | 12 | Page11-13 (Line205, Line249) |
| Participant timeline | 13 | Page5 (Line110)  Page13 (Line250) |
| Sample size | 14 | Page8 (Line142) |
| Recruitment | 15 | Page7 (Line134) |
| **Methods: Assignment of interventions (for controlled trials)** | | |
| Allocation: |  |  |
| Sequence generation | 16a | Page8 (Line155) |
| Allocation concealment mechanism | 16b | Page8 (Line156-157) |
| Implementation | 16c | Page8-9 (Line157-161) |
| Blinding (masking) | 17a | Page9 (Line163-167) |
|  | 17b | Page9 (Line167-170) |
| **Methods: Data collection, management, and analysis** | | |
| Data collection methods | 18a | Page13 (Line250-267) |
|  | 18b | Page18 (Line348-349) |
| Data management | 19 | Page14 (Line258-267) |
| Statistical methods | 20a | Page14 (Line274-279) |
|  | 20b | Page15 (Line280-282) |
|  | 20c | Page14 (Line270-273) |
| **Methods: Monitoring** | | |
| Data monitoring | 21a | Page15 (Line290-301) |
|  | 21b | Page15 (Line294-301) |
| Harms | 22 | Page15 (Line283-289) |
| Auditing | 23 | Page14 (Line259-267), Page15 (Line292-294) |
| Ethics and dissemination | | |
| Research ethics approval | 24 | Page5 (Line106-119) |
| Protocol amendments | 25 | Page15 (Line294-296) |
| Consent or assent | 26a | Page5 (Line104) |
|  | 26b | Not applicable. No biological samples or other additional samples. |
| Confidentiality | 27 | Page13-14 (Line258-266) |
| Declaration of interests | 28 | Page19 (Line378-379) |
| Access to data | 29 | Page14 (Line259-265) |
| Ancillary and post-trial care | 30 | Page15 (Line285-287) |
| Dissemination policy | 31a | Page18 (Line367-369) |
|  | 31b | Page19 (Line380-382) |
|  | 31c | Page18 (Line367-369) |
| Appendices |  |  |
| Informed consent materials | 32 | Page5 (Line103-105) |
| Biological specimens | 33 | Not applicable. No biological specimens. |

*It is strongly recommended that this checklist be read in conjunction with the SPIRIT 2013 Explanation & Elaboration for important clarification on the items. Amendments to the protocol should be tracked and dated. The SPIRIT checklist is copyrighted by the SPIRIT Group under the Creative Commons “[Attribution-NonCommercial-NoDerivs 3.0 Unported](http://www.creativecommons.org/licenses/by-nc-nd/3.0/)” license.
