# Supplementary material for: Auricular acupressure for myopia prevention and control in children and its effect on choroid and retina: a randomized controlled trial protocol
Source: Trials. 2021 Jun 7;22:387. doi: 10.1186/s13063-021-05334-1 (PMC8186104; doi:10.1186/s13063-021-05334-1)
Supplement: Supplementary file 5 — Additional file 5. All items from the World Health Organization Trial Registration Data Set [file 13063_2021_5334_MOESM5_ESM.docx]

All items from the World Health Organization Trial Registration Data Set

| **Data category** | **Information** |
| --- | --- |
| Primary registry and trial identifying number | ChiCTR2000038456 |
| Date of registration in primary registry | 23 Sep, 2020 |
| Secondary identifying numbers | ChiMCTR2000004185 |
| Source(s) of monetary or material support | Shanghai Municipal Health Committee |
| Primary sponsor | Shuguang Hospital Affiliated to Shanghai University of Traditional Chinese Medicine |
| Secondary sponsor(s) | Shuguang Hospital Affiliated to Shanghai University of Traditional Chinese Medicine |
| Contact for public queries | Rong HAN (HanR1994@163.com) |
| Contact for scientific queries | Rong HAN (HanR1994@163.com); Feng ZHAO (doczhaofeng@163.com) |
| Public title | Clinical Effect of Auricular Acupressure on Prevention and Control of Myopia and Its Influence on Choroid and Retina |
| Scientific title | Clinical Effect of Auricular Acupressure on Prevention and Control of Myopia and Its Influence on Choroid and Retina |
| Countries of recruitment | China |
| Health condition(s) or problem(s) studied | Myopia |
| Intervention(s) | Intervention group: auricular acupressure(six acupoints, press three times a day). |
|  | Control group: no intervention. |
| Key inclusion and exclusion criteria | Inclusion criteria: ages 8 to 9 years old; SE (after cycloplegia): -0.5D - +0.5D; uncorrected visual acuity ≥0.8; corneal curvature: 40D-46D; with good compliance, and the participant and his/her guardians signed the informed consent form. |
|  | Exclusion criteria: combined with other eye diseases or systemic diseases; with active eye diseases or had a history of eye surgery; those who have used atropine, orthokeratology lens or other myopia prevention and control methods; skin lesion at the to-be-treated auricular acupoints or sensitive to the ear adhesive; subjects who cannot cooperate to complete the treatment; the guardian has unreasonable expectation. |
| Study type | Interventional study |
|  | Allocation: randomized; Intervention model: parallel assignment |
|  | Primary purpose: prevention. |
| Date of first enrolment | Oct 2020 |
| Target sample size | 480 |
| Recruitment status | Recruiting |
| Primary outcome(s) | Myopia incidence and spherical equivalent. |
| Key secondary outcomes | Axial length, uncorrected visual acuity and choroidal and retinal thickness |
